# AVISNet: An edge-computing model for acoustic identification of species from the Doñana bird community

**DOI:** 10.1371/journal.pone.0360378

**Published:** 2026-11-04

**Authors:** Delia Velasco-Montero, David Solís-Martín, Jorge Fernández-Berni, Giulia Bastianelli, Javier Bustamante

**Affiliations:** 1 Instituto de Microelectronica de Sevilla (IMSE-CNM), Universidad de Sevilla-CSIC, Seville, Spain; 2 Universidad de Sevilla, Dpto. Ciencias de la Computación e Inteligencia Artificial, Sevilla, Spain; 3 Estacion Biologica de Doñana (EBD), CSIC, Seville, Spain; 4 ICTS-Doñana, Estacion Biologica de Doñana (EBD), CSIC, Seville, Spain; Georgia State University, UNITED STATES OF AMERICA

## Abstract

The global decline in biodiversity calls for scalable and efficient species monitoring. Birds, as sensitive ecological indicators with rich vocal activity, are particularly well suited for passive acoustic monitoring (PAM), which enables extensive, non-invasive data collection. Yet the large volumes of audio generated by PAM require automated and efficient processing pipelines to extract meaningful biological information in real time. To address this challenge, we integrated automated acoustic species identification directly into embedded devices (i.e., *at the edge*), enabling autonomous on-site data processing without reliance on cloud-based computation. Our methodology emphasizes model efficiency and leverages lightweight pre-trained architectures (e.g., MobileNet), alongside advanced learning strategies such as knowledge distillation and few-shot learning to overcome data scarcity for under-represented species. This methodology was applied as a case study to acoustic identification of bird species native to Doñana National Park, Spain. We assessed the performance and efficiency of our models at three levels of acoustic data: focal segments, focal recordings, and soundscape recordings. On focal recordings, our models achieve up to 0.70 F1-score in species identification task. At the soundscape level, we evaluated performance using three real-world soundscape datasets, measuring accuracy metrics at both the vocalization and dataset levels. Our lightweight convolutional neural networks achieve high accuracy at the dataset level (F1-score: 0.56–0.72), solid accuracy at the vocalization level (F1-score: 0.29–0.39; mAP: 0.41-0.69), and fast and energy-efficient inference on resource-limited hardware (2.2–4.7× speed-ups vs. BirdNET, 41–71% energy savings). Overall, our results show that these networks achieve functional species recognition performance while substantially reducing inference latency and energy requirements relative to BirdNET, thus enabling on-device deployment on low-power units. The system already scales to 300 + species, with room for further expansion, bridging the gap between general-purpose tools like BirdNET and localized embedded monitoring pipelines.

## 1. Introduction

The accelerating decline of global biodiversity presents a critical challenge for conservation, necessitating rapid and effective monitoring of species across large spatial and temporal scales [1,2]. Passive acoustic monitoring (PAM) [3] has emerged as a non-invasive method to track wildlife over extended periods through audio recordings. Networks of field-deployed autonomous recording units (ARUs) now enable the systematic collection of vast acoustic datasets across diverse habitats [4–6]. However, extracting ecologically meaningful information from these data remains a major bottleneck, particularly for real-time and on-site processing [7]. Advances in artificial intelligence (AI) offers significant promise in addressing these challenges, with its ability to analyze large datasets and automate species identification tasks [8,9]. A persistent challenge of AI tools for ecology is that most of them are designed for operation on off-site, high-performance computing systems, requiring periodic retrieval of data from field devices for subsequent processing [10]. This approach is resource-intensive, laborious, and costly, limiting its scalability and sustainability. For instance, processing acoustic data requires frequent retrieval of data from field-deployed ARUs – as SD cards must be replaced regularly due to memory constraints – which is costly and limits monitoring scalability.

Therefore, to enable more efficient and scalable monitoring, there is a pressing need to develop lightweight AI models that can be deployed directly on field devices. Such models would enable autonomous, on-site data analysis, which (i) reduces the need for manual intervention, (ii) supports real-time decision-making, and (iii) allows to extend the life of SD card memories by eliminating irrelevant data [11,12]. Real-time or near-real-time species identification at the edge can provide substantial benefits in several ecological monitoring scenarios. For instance, in protected areas, the detection of threatened species during sensitive periods such as breeding may enable managers to temporarily restrict visitor access to specific locations, reducing the risk of disturbance while the biological event is occurring. When communication infrastructure is available, these detections can support rapid management responses and targeted field actions by conservation staff and other stakeholders. Real-time information may likewise benefit ecotourism and birdwatching activities by informing visitors about the recent presence of rare or highly sought-after species in locations where their occurrence is otherwise difficult to predict. Another important application concerns autonomous monitoring networks deployed in remote areas. Transmitting complete audio recordings often requires considerable communication bandwidth and energy resources, or alternatively periodic retrieval of storage media, which increases operational costs. By performing species identification directly on the recording device and transmitting only the resulting detections and metadata, communication requirements can be substantially reduced. This reduction can facilitate longer deployments, more frequent monitoring schedules, or operation in locations where network connectivity is limited. These considerations are particularly relevant in large protected areas such as Doñana National Park, where monitoring stations may be distributed across extensive and environmentally sensitive habitats. The relevance of these advantages depends strongly on the monitoring strategy. In some PAM studies, duty-cycled recording schedules can substantially reduce storage and energy requirements, making long deployments feasible without frequent maintenance. However, in continuous-monitoring applications, large-scale sensor networks, or deployments in remote locations, the ability to perform on-device species identification can provide additional benefits, including near-real-time ecological information, reduced communication requirements, and lower operational costs associated with storing, transmitting, or retrieving large volumes of audio data.

### 1.1. Related work

In ecoacoustic research, deep learning has become a powerful tool for processing and analyzing bird vocalizations. The standard pipeline involves three main stages: preprocessing, feature extraction, and automatic recognition [13–15]. The most frequent tasks are species classification and detection, with convolutional neural networks (CNNs) dominating due to their superior performance over traditional approaches [14]. Most architectures operate on spectrogram representations of short audio segments (1–10 seconds) using small convolutional filters, often employing Mel-scaled frequency axes [16]. Beyond CNNs, convolutional recurrent networks (CRNNs) and transformer-based architectures have been proposed for bird sound classification [17,18]. While CNNs can effectively learn spectro-temporal patterns directly from spectrogram representations, CRNNs and transformers explicitly model longer-range temporal dependencies and sequential context, which may further improve species identification performance in complex acoustic environments. When labeled data are scarce, alternative strategies such as unsupervised clustering (e.g., via autoencoders), or semi-supervised learning leveraging labeled subsets for feature extraction, have been explored [19]. Data augmentation is widely employed to improve robustness, using transformations such as time shifts, background noise addition, mixing, or spectrogram warping [14,20]. For model initialization, transfer learning from vision (ImageNet) is occasionally used, as well as pretraining on large-scale audio datasets like Google AudioSet [21] or VGG-Sound [22].

The most commonly used approach for automated identification of birds is BirdNET [23], which covers a large number of species from North America and Europe and demonstrates competitive performance. It is a deep residual CNN [24] with 157 layers and over 27 million parameters, trained on more than 6,500 species. BirdNET, which was trained using extensive augmentation, processes 3-second Mel spectrograms. Despite its high performance and adoption, several limitations are documented:

- Threshold dependence: detection accuracy varies widely across species, requiring species-specific confidence thresholds to balance sensitivity and specificity [25–29]. Default threshold (0.1) often yield high false-positive rates, while strict values risk false negatives [26,27]. Studies recommend optimized thresholds (e.g., 0.3–0.65 depending on F-score weighting) for better performance [27,30–32].
- Calibration issues: BirdNET’s raw scores cannot be directly interpreted as a strict probability of species presence [33]. To mitigate this, techniques such as logistic regression can improve alignment with real-world detection probabilities.
- Species coverage gaps: regional species of interest – in our case local to Doñana National Park, hereafter referred to as Doñana N. P. – may not be included in the model.

BirdNET has also been leveraged through transfer learning and embedding extraction, reducing the need for large labeled datasets. For example, embeddings have been successfully used for coral reef soundscapes [34], for classifying owl call types [35], and in other processing pipelines [36]. These approaches highlight BirdNET’s value as a feature extractor, even outside its original scope.

Concerning embedded audio-data processing, research remains limited. Some efforts explored CNNs for environmental sound recognition on field-programmable gate arrays (FPGAs) and tensor processing units (TPUs), achieving high accuracy but at the expense of costly, specialized hardware [37]. Alternative approaches such as Bird@Edge demonstrated the feasibility of efficient CNN architectures on embedded devices with high recognition performance [38]. Networks of low-cost acoustic sensors with distributed processing have also been proposed for urban monitoring [39]. Further studies show successful CNN deployment on Raspberry Pi (RPi) devices with inexpensive microphones, sometimes integrated with LoRa or WiFi modules for communication [40,41]. Evaluations of lightweight classical ML techniques confirm the trade-offs between accuracy, latency, and energy consumption in edge audio processing [42]. However, most of these approaches are either limited to urban sound categories or rely on small-scale classification tasks, leaving the challenge of broad, species-rich ecological monitoring largely unaddressed.

Finally, in the context of models for local species, authors in [43] employed a CNN with four convolutional layers to recognize 101 bird species using local data from a region in Southern Finland, while HawkEars [44] was trained for Canadian species. Specifically for Doñana N. P., recent work [45] proposed a two-stage pipeline that combines a bird song detector with fine-tuned classifiers, targeting 34 local species. However, broader approaches covering the full community of Doñana’s biodiversity remain unexplored. Such regional models suppose an emerging attractive paradigm, because local models can reduce the computational requirements and the classification space, while remaining focused on the ecological objectives of a specific monitoring program. Localized models may also offer greater flexibility for future extensions tailored to specific monitoring objectives, such as distinguishing among different vocalization types within a species or incorporating region-specific ecological requirements.

To address this challenge, this work introduces AVISNet (Acoustic Vocalization Identification Network for avian Species), a family of lightweight CNNs specifically designed for the audio-based identification of more than 300 bird species associated to Doñana N. P. (SW Spain), one of Europe’s most important bird biodiversity hotspots [46]. Doñana’s ecological significance, together with the presence of species underrepresented or absent from global recognition systems such as BirdNET, motivated the development of a tailored acoustic classification model. AVISNet is optimized for embedded deployment on ARU devices, enabling autonomous, energy-efficient on-site analysis with the advantages outlined above. Our main contributions are as follows:

- A novel methodology combining advanced learning techniques such as few-shot learning and knowledge distillation to effectively integrate rare species with limited recordings into the classification model.
- A ready-to-use lightweight bird species classifier tailored for Doñana N. P., including species not available in BirdNET, as well as different versions of the model.
- Released code and weights, in line with FAIR principles (Findable, Accessible, Interoperable, Reusable), to facilitate reuse by the ecological and AI communities.
- Model benchmarking in terms of (i) classification accuracy and (ii) inference performance.

Rather than competing directly with large-scale global classifiers, this work investigates whether a compact regional model can provide a useful trade-off between computational efficiency and species-recognition performance for local PAM applications. The underlying hypothesis is that constraining the classification task to the bird community of a specific region – thus including regional species that general-purpose global tools omit – may enable practical deployment on resource-limited devices while maintaining sufficient performance for ecological monitoring purposes.

## 2. Materials and methods

### 2.1. Doñana bird community

This study focuses on Doñana N. P. (SW Spain), one of Europe’s most important biodiversity hotspots [46]. Its strategic location (Fig 1) along major migratory flyways makes it a key stopover and wintering area for thousands of birds each year. Our target species list, comprising 336 bird species, was curated by ornithologists specialized in the bird community of Doñana. The list includes all species commonly observed within Doñana N. P., excluding rare or irregular occurrences. Of these, 14 species are not included in BirdNET’s taxonomy, underscoring the need for a localized recognition system.

**Fig 1:**
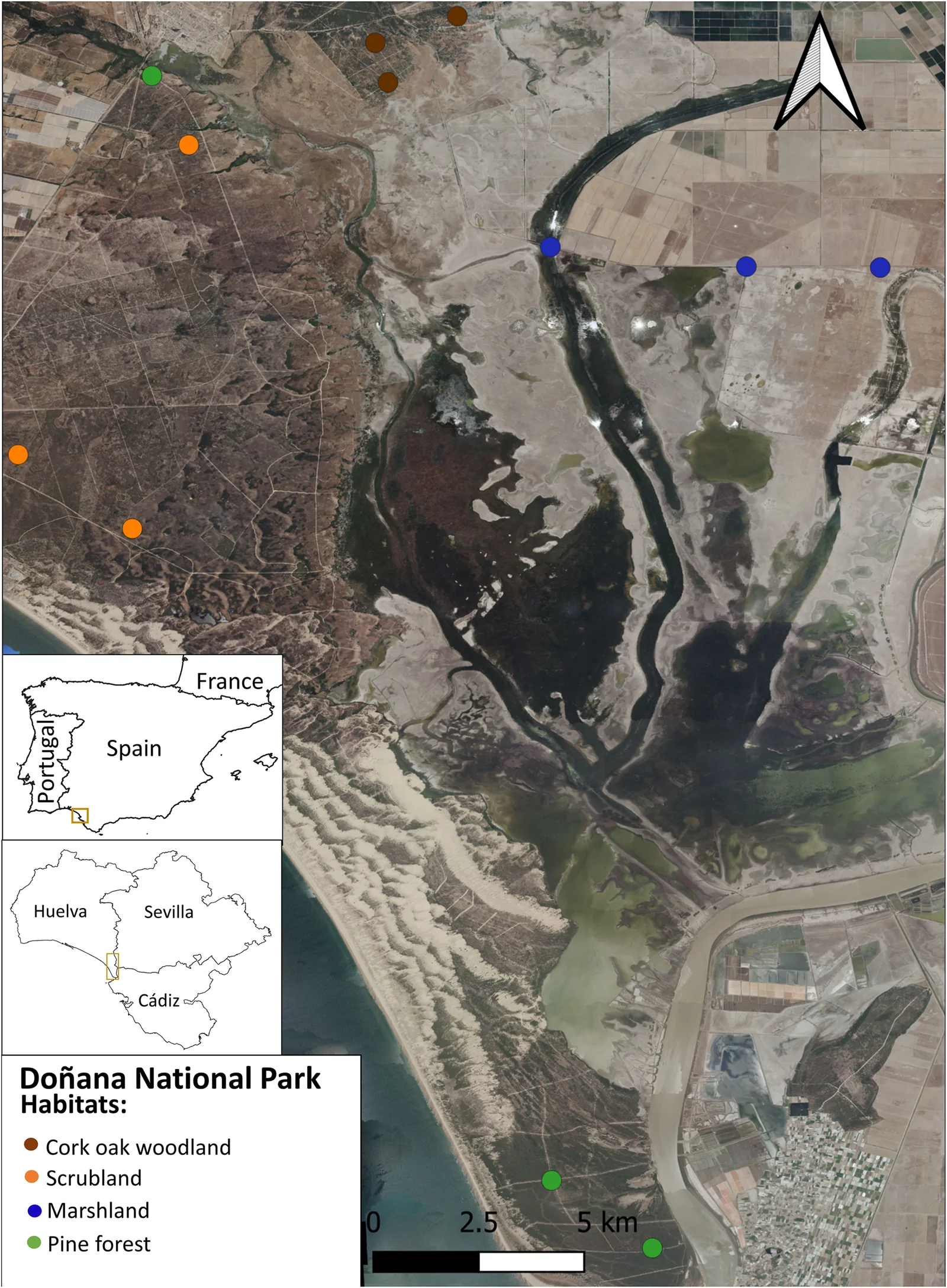
Doñana National Park and its geographic location within the Iberian Peninsula. The colored points represent the spatial distribution of the 12 sampling sites belonging to the Doñana2425 dataset (see Section 2.6), according to their respective habitat. The map was produced using QGIS v.3.28.3, with orthophoto imagery from the Spanish National Aerial Orthophotography Plan (PNOA) used as the background layer.

### 2.2. Data collection for model training

We compiled a training dataset from multiple bioacoustic platforms to maximize recording diversity, vocal variations, and variability in recording quality [15]. Xeno-Canto (XC) [47] was the primary source, accessed via XC API [48]. Given that filtering by Spain provided only partial coverage (leaving 58 species unrepresented), we collected recordings without applying any geographic restriction, which ensured coverage of all target species. To avoid extreme class imbalance, a maximum of 1,000 files per species was retained. We also included recordings from the iNaturalist Sounds platform (iNat) [49] as a complementary source, covering most but not all species (61 absent). We had to harmonize the scientific names from XC and iNat because taxonomic updates and spelling variants are common in bioacoustic repositories. For robust inference, we also included a ‘other’ class covering non-bird sounds (human activity, environmental noise, domestic/exterior noises, music). These were drawn from AudioSet [21], a large-scale dataset of audio events, and ESC-50 [50], a curated collection of environmental sounds, ensuring the model could discriminate background and anthropogenic sounds from relevant bird vocalizations.

### 2.3. Training data curation and preparation

**Audio segmentation and strong labeling** Training data for CNNs should consist of spectrogram images generated from *n*-second audio intervals. Collected recordings are weakly labeled, providing species-level annotations without temporal boundaries, given that strong labeling (including annotation timestamps) is prohibitively expensive and rarely available [51]. Consequently, labels correspond to the entire file, even though birds do not vocalize continuously and recordings may contain silence or background noise. Therefore, direct segmentation into fixed-length temporal windows for training, risks including segments devoid of target vocalizations, which can degrade model training.

To address this and mitigate the effects of silent or noisy intervals, several strategies were considered: fixed-length segmentation, decibel-thresholding, spectrogram-based thresholding or region selection [52], and BirdNET-assisted labeling. Preliminary experiments comparing these methods showed us that BirdNET-assisted segmentation yielded the most effective results. Accordingly, we adopted this strategy of applying BirdNET-assisted pseudo-labeling to generate strong labels for the weakly-labeled recordings from XC and iNat. This means we retained segments with high-confidence predictions (≥ 0.5) whose detected species matched the ground truth label, while discarding others; as shown in Fig 2. This approach reduces label noise and improves dataset quality, under the assumption that BirdNET’s high-confidence predictions are likely correct. Pseudo-labeling is commonly used in ecoacoustic applications where manual labeling is costly [53–55]. Notwithstanding, this technique results in a training dataset which is skewed toward high-SNR or ‘acoustically clean’ examples. While this commonly-used strategy effectively prevents the network from learning from noisy or silent windows, it also favors relatively easy vocalizations. Generalization to complex soundscapes remains fundamentally constrained by the representativeness of the filtered training data. For the 14 species not included in BirdNET, segmentation was instead performed using spectrogram-based region selection with scikit-maad library [56], a Python toolkit for ecoacoustic analysis, to detect prominent spectral regions, followed by manual inspection to discard residual silence or noise.

**Fig 2:**
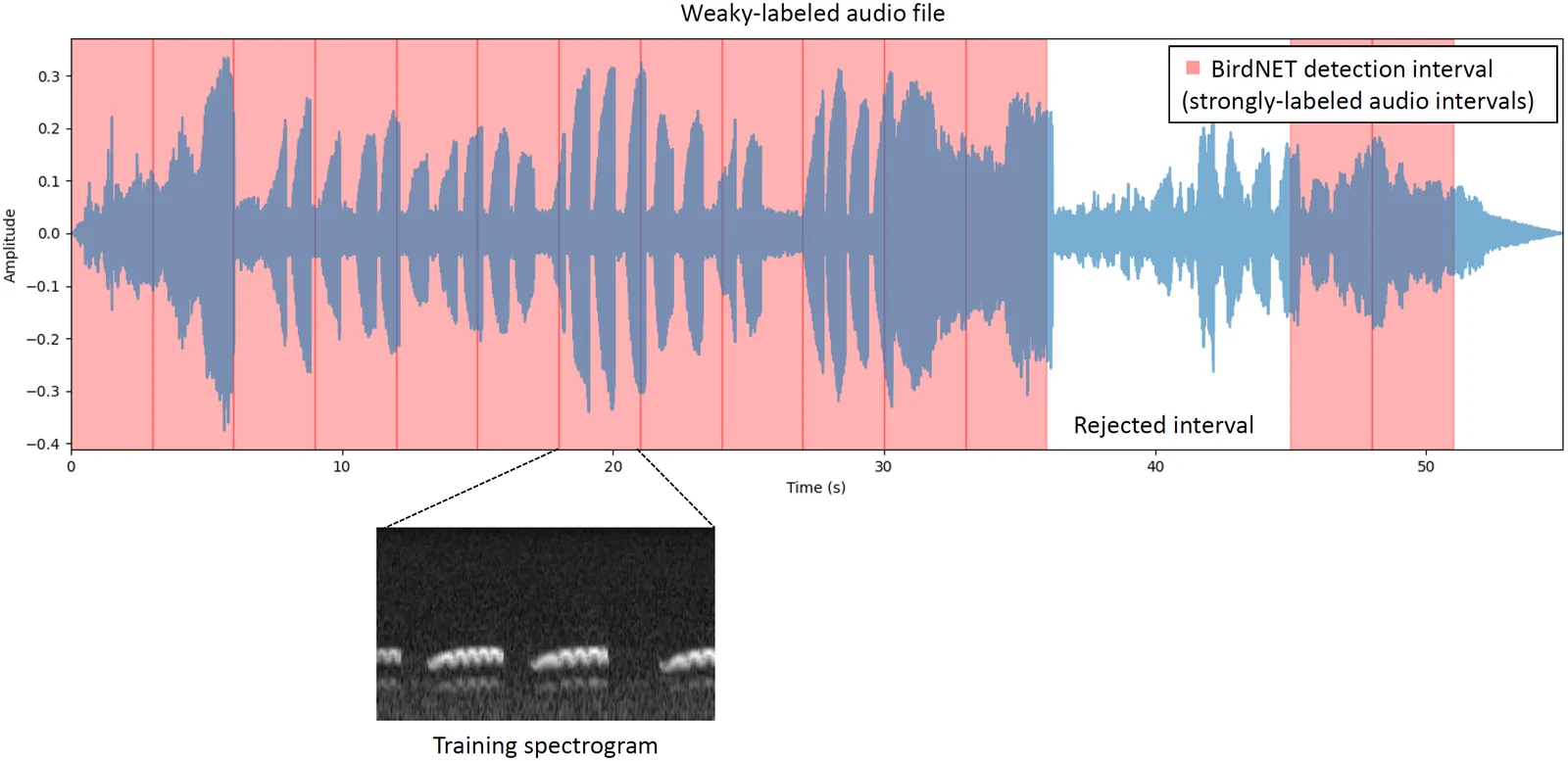
Overview pf the preprocessing pipeline. *Top*: Audio segmentation based on BirdNET-assisted pseudo-labeling, with retained segments highlighted. *Bottom*: Spectrogram generation for each extracted 3-second interval.

**Dataset split and data balance** The collected dataset comprised over 200k audio files, totaling 480 GB. Data segments were stratified into training, validation, and test sets, ensuring that segments from the same recording remained within a single subset to prevent data leakage. A 60–20–20 split was applied, consistent with the iNat dataset’s original partitioning. To address class imbalance while accounting for hardware limitations during training, a maximum of 1,000 randomly selected segments per species and subset was retained.

**Spectrogram generation** Spectrograms were generated following a parameterization inspired by BirdNET, which has been shown to perform well for bird vocalizations. Our model uses 3-second windows – aligned with the 2-second average duration of human-annotated bird vocalizations [23] –, together with a restricted frequency range between 150 Hz and 15 kHz, and Mel compression with a break frequency at 1,750 Hz. Since our models leverage CNNs pre-trained on ImageNet, which typically expect 224×224 inputs, we needed to adjust parameters to increase frequency resolution as much as possible while maintaining temporal precision. Specifically, we set 1024 samples for the FFT algorithm (*NFFT* = 1024) and 128 Mel bands, producing spectrograms of 128×224. To ensure compatibility with the input layer of the CNN, these spectrograms are dynamically resized on-the-fly to a standard resolution of 224×224 during the runtime data-loading process, using high-quality bicubic interpolation (which smoothly preserves bioacoustic syllable boundaries). An example spectrogram is shown in Fig 2.

**Data augmentation** To improve model generalization and robustness to real-world acoustic variability, spectrograms were augmented during training using standard transformations for audio classification [43]. These include time- and frequency-domain manipulations (stretching, shifting, masking), intensity adjustments, and additive noise. Augmentations were applied on-the-fly to training batches.

### 2.4. Model training

Models were trained using the open-source machine learning library TensorFlow [57] version 2.14.1, running on a high-end computer under Linux Ubuntu 22.04.5, employing a dataset composed of more than 600k images.

Because the models are intended for embedded devices, we adopted the MobileNet family [58,59] as backbone architecture, which employs depthwise separable convolutions to greatly reduce computation. MobileNet offers additional flexibility via width (α) and resolution (ρ) multipliers, allowing trade-offs between efficiency and accuracy. These characteristics, along with the availability of ImageNet-pretrained weights for different model sizes and input resolutions, motivated our choice. The backbone was adapted by replacing the 1000-class fully connected layer with a global average pooling layer followed by two dense layers (128 units and number of output classes, respectively).

Preliminary experiments showed us that training on categories with limited data tends to overfitting. Therefore, our initial training set was composed of well-represented categories (Fig 3-left), with under-represented classes added later via few-shot learning (FSL) techniques (Fig 3-right). Notably, species absent from BirdNET often correspond to categories with scarce data.

**Fig 3:**
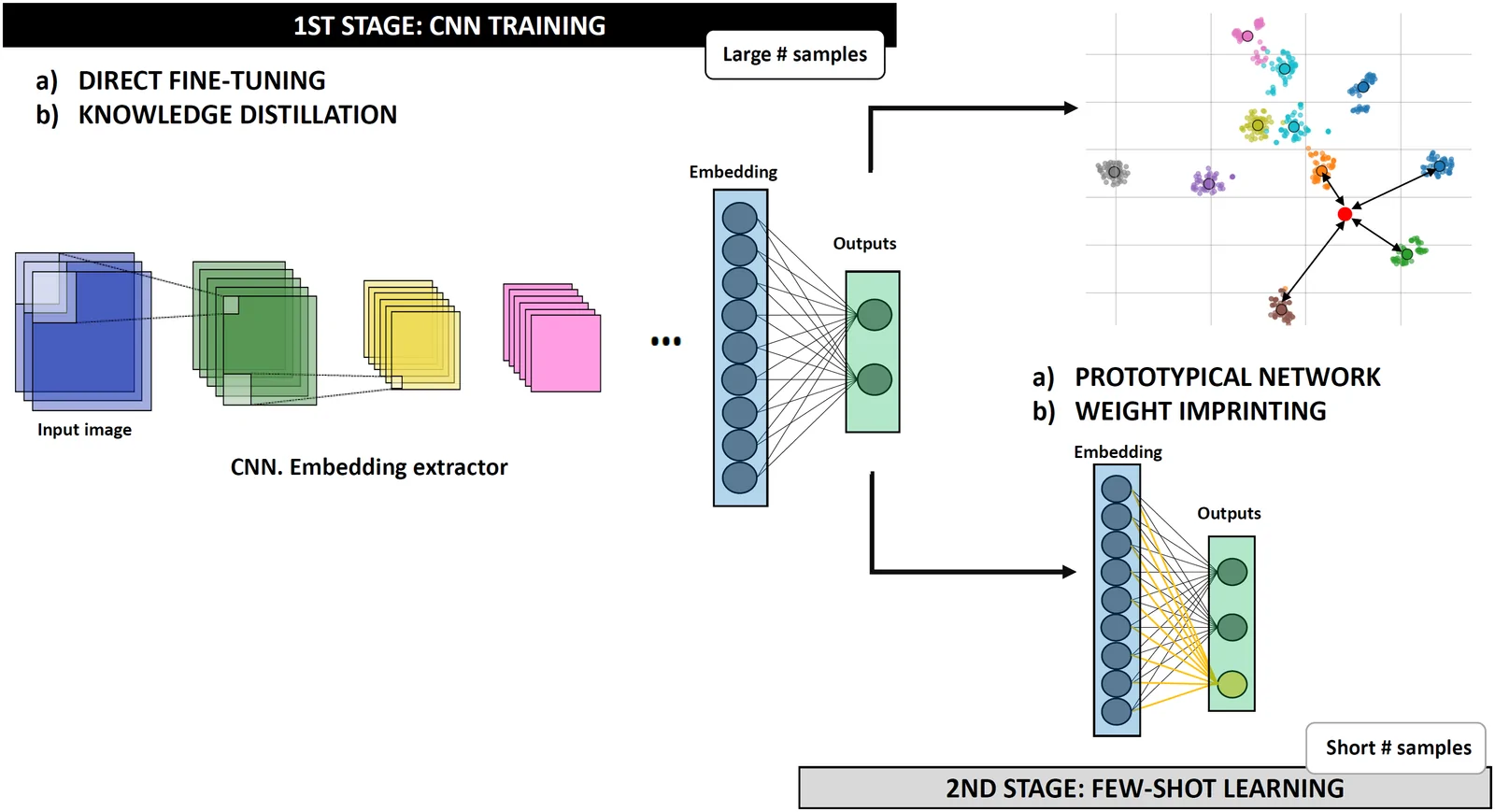
Training workflow: in the first stage, the CNN is trained on well-represented classes; in the second stage, rare species are incorporated using FSL.

#### 2.4.1. First stage: Fine-tuning and knowledge distillation.

Two complementary strategies were explored for well-represented classes: (a) direct supervised training using labeled spectrograms and ImageNet-pretrained weights, and (b) knowledge distillation (KD) [60] on pretrained weights using BirdNET as teacher. KD allows the student model to learn from both hard labels and soft predictions from the teacher, capturing inter-class relationships and improving generalization. This typically results in models which are particularly suitable for resource-constrained devices. To obtain the soft labels, BirdNET scripts were modified to extract the prediction vectors for each audio segment. A temperature parameter *T* softens the teacher outputs, and the total loss is an α-weighted combination of categorical cross-entropy (hard labels) and Kullback-Leibler divergence (soft labels).

In this first stage (Fig 3-left), we trained the MobileNet architecture with two input resolutions (224×224 and 128×128). Only categories meeting the minimum data requirement (50 images) were included, resulting in 305 classes. Training followed a two-stage approach: first, only the classification head was trained for 10 epochs; second, the entire model was fine-tuned. The Adam optimizer was used with an initial learning rate of $5\times 10^{-4}$ (exponentially decayed every 2 epochs), early stopping, dropout of 0.3, batch size of 32, and T=2,α=0.5 for KD.

#### 2.4.2. Second Stage: Few-shot learning for under-represented species.

After training the CNN on well-represented species, our methodology employs FSL [61] to incorporate under-represented species. Two FSL strategies were explored:

- (a) Prototypical networks [62] (Fig 3-left): the CNN acts as an embedding extractor $\begin{array}{c} f_{\theta }(x) \end{array}$ with learnable parameters $\begin{array}{c} \theta \end{array}$ that maps input data ***x*** to a ***d***-dimensional feature space. This approach represents each class by a prototype in a learned embedding space, ${𝐜}_{k}$ for each class k∈{1,...,C}. Class prototypes are computed as the mean of available sample embeddings. At inference time, classification is performed by assigning each input sample to the nearest prototype in the Euclidean space. The probability of belonging to class *k* is computed via a softmax over the negative distances to each class prototype.
- (b) Weight imprinting [63,64] (Fig 3-right-bottom): this approach considers that each row of the weight matrix of the final fully-connected (classification) layer is similar to its class prototype in the embedding space. Therefore, the average embedding vector of the available samples for a new class $\begin{array}{c} f_{\theta ,avg}({𝐱}_{i}) \end{array}$ can be directly “imprinted” into the weight matrix – along with a normalization function $\begin{array}{c} F(f_{\theta ,avg}({𝐱}_{i}),w_{i,avg}) \end{array}$. This provides a strong starting point for rare classes (model can then be fine-tuned), while retaining performance on well-represented classes.

All trained models, scripts, and documentation required to run the networks are available in a public GitHub repository [65].

### 2.5. Evaluation methodology

To ensure a fair and comprehensive assessment of AVISNet, we consider different data sources, levels of analysis, common evaluation metrics, and comparisons against a widely-used baseline model (BirdNET):

1. **Types of audio data** In bioacoustics we can distinguish between *focal recordings*, which typically feature a foreground subject, and *soundscapes* or *passive recordings*, which often contain overlapping vocalizations from multiple species in the background [66]. We first evaluate the models on a subset of the training data composed of focal recording (XC and iNat, Section 2.2); we then assess performance on real-world soundscapes (Section 2.6), which provide a more challenging and ecologically realistic test of model robustness.
2. **Levels of evaluation** We distinguish between two complementary levels of evaluation [27,29]:
  - At the *vocalization level*, models are tested on individual bird vocalizations, by evaluating model predictions at the corresponding timestamps (this requires strong annotations). In this context, we define a True Positive (TP) vocalization when the predicted species matches a ground-truth species in the prediction interval, False Positive (FP) when a species is predicted but absent from the ground truth, and False Negative (FN) when a ground-truth species exists but is not correctly predicted. True negatives (TN) are not defined in this setup [29].
  - At the *dataset level*, we compare the set of species present with the set detected by the model. Then, the same metrics as those of vocalization level can be defined. This broader view provides insights into community composition and enables ecological indicators such as species richness.
3. **Model performance metrics** Model performance was evaluated at both vocalization and dataset levels, using standard multi-class classification metrics: **Precision** (proportion of correct positive predictions among all positive predictions), **recall** (the proportion of actual positives correctly identified by the model), and **F1-score** (the harmonic mean of precision and recall) were employed to evaluate all classes, giving equal weight to both frequent and rare species (‘macro’ metrics). **Top-1 accuracy** was computed for focal recordings, where each audio file predominantly contains a single species. For soundscape recordings, where multiple simultaneous vocalizations may occur and segments can contain more than one species, top-1 accuracy is less representative and can be misleading. **Mean Average Precision** (mAP, which summarizes overall precision-recall performance across confidence thresholds for all classes) is reported at the level of individual detections (i.e., vocalization events), as it directly summarizes the quality of prediction scores. Finally, the number of correct species detected at the dataset level (dataset-level TP) is also reported, since it provides biologically meaningful insights into community composition.
4. **BirdNET comparison** As a baseline, we compare our models against BirdNET using its recommended configuration for ecological soundscape analysis. Following recent studies [27,32], we used a 2-second overlap, a detection sensitivity of 1.5, and confidence thresholds of 0.3 at the vocalization level and 0.5 at the dataset level.

### 2.6. Real-world soundscape evaluation datasets

To evaluate model performance under realistic ecological conditions, we additionally tested our models on three real-world soundscape datasets, with detailed results provided in Section 3.3. These datasets differ in size, species composition, habitat coverage, and recording conditions.

**Doñana2425 [**67**]** Between December 2024 and May 2025, we manually annotated 5-minute field recordings collected across 12 sampling sites representing the main macrohabitats of Doñana N. P. (Fig 1): scrubland, cork oak woodland, marshland, and pine forest. Each recording was labelled to obtain the list of species detected within its 5-minute duration. The resulting dataset comprises 72 annotated recordings, covering 81 species (all included in AVISNet), whose distribution is shown in Fig 4. The evaluation process is conducted at the temporal scale of the 5-minute recording, instead of vocalization recording, using the species list derived for each file. Such temporal scale is appropriate for characterizing the bird community present at each site [68,69].

**Fig 4:**
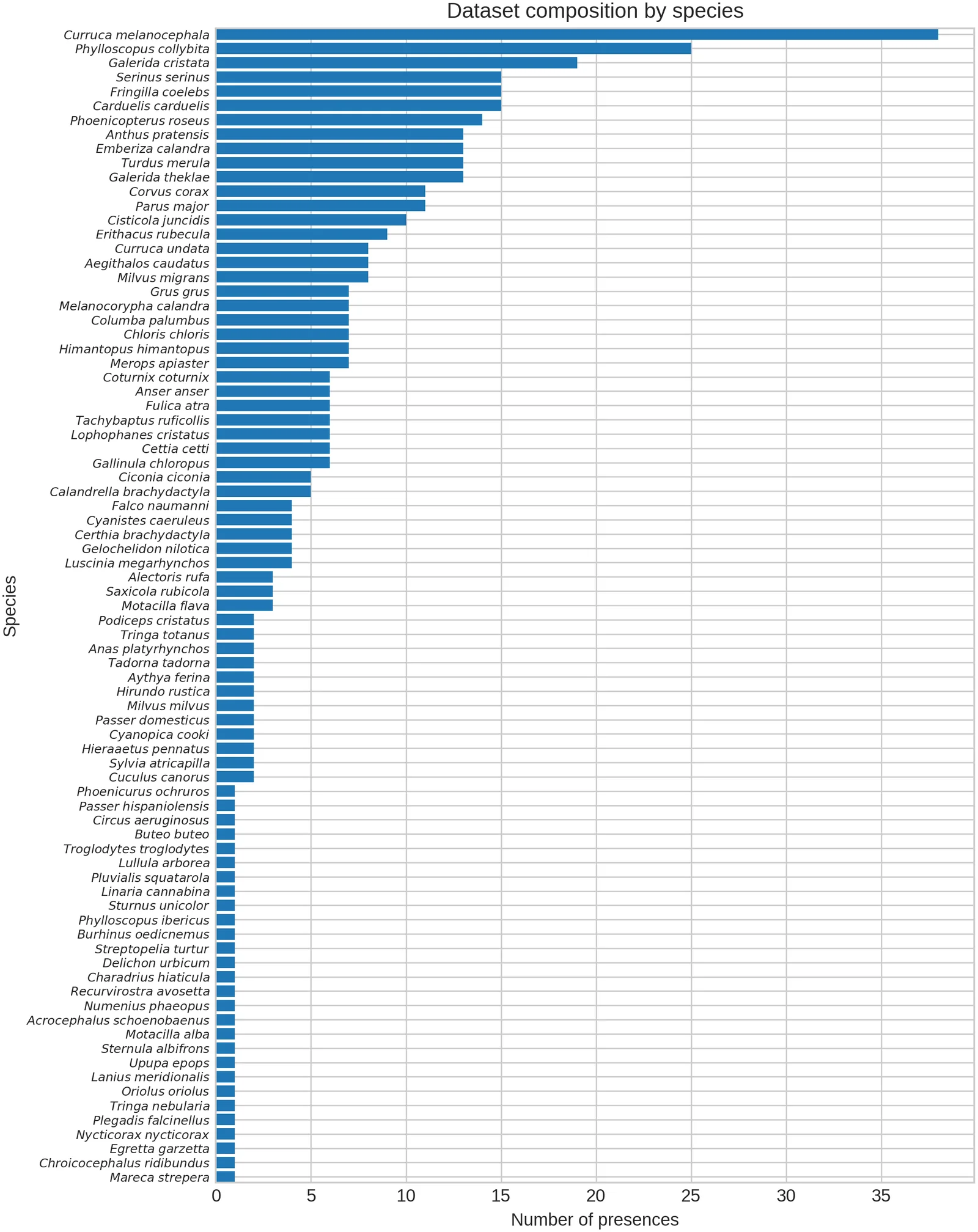
Species composition of annual census in Doñana2425 dataset.

**WABAD [**70**]** This collection comprises recordings of 1,147 bird species collected at 70 sites across 27 countries and 13 different biomes. Given its broad global scope, we restricted our analysis to those recordings containing species from our reference list of 336 species of interest in Doñana. This filtering resulted in 1,853 recordings from 40 sites, all with temporal annotations. Within this dataset, 161 species overlap with our target species list, making this dataset particularly useful for evaluating generalization capability of models across heterogeneous conditions at both space (regions, habitats) and time (season, time of day) [29].

**NIPS4BPlus [**71**]** This dataset consists of soundscape recordings collected in Spain, Southern France, and Central France. It includes 674 individual recordings with detailed temporal annotations (training subset). A total of 88 categories are represented, including various corresponding to insects and amphibians. Out of these, 45 bird species overlap with the reference list of 336 species of interest in Doñana N. P.

## 3. Results and discussion

### 3.1. Performance on focal-recording segments

In the first stage (Section 2.4.1), two flavors of MobileNet (224×224, 128×128) were trained using two approaches: direct supervised training and KD using BirdNET as teacher. When testing on the testing subset of focal-recording audio segments, the model trained on 305 well-represented species at 224×224 achieved top-1 accuracy of 0.94, F1-score of 0.91, and mAP of 0.95 with direct training, slightly outperforming KD (0.93, 0.90, 0.95, respectively). At 128×128 resolution, both approaches reached 0.90, 0.85, and 0.91, respectively. This similar performance observed between direct training and KD, is likely due to the use of pseudo-labeled segments for training (Section 2.3). We anticipate that if alternative audio-noise segmentation methods are employed, KD could enhance convergence.

Then, the FSL techniques described in Section 2.4.2 were applied to expand the classification capacity to 337 classes (336 species plus the ‘other’ class). Performance after applying both FSL methods – prototypical networks and weight imprinting with a 3-epoch fine-tuning – is reported in Table 1. Weight imprinting consistently outperforms prototypical networks across input resolutions. A key advantage of this approach is that it preserves the performance of previously trained categories, while prototypical networks may alter decision boundaries and degrade accuracy on well-represented classes. Therefore, in the remainder of this work we refer to these resulting models employing weight imprinting as *AVISNet-224* and *AVISNet-128*. A deeper analysis reveals that, as expected, the lowest-performing categories correspond to species with the least available training data. This underscores the trade-off between expanding the classification scope and maintaining high accuracy on under-represented species [27].

**Table 1:** Performance on focal-recording segments^*a*^ for FSL methods (2nd stage) after feature extractor training (1st stage).

Method	Input resolution	Top-1 acc.	F1-score	mAP
(a) Prototypical Network	224×224	0.80	0.76	0.80
(b) Weight Imprinting	224×224	**0.94**	**0.84**	**0.89**
(a) Prototypical Network	128×128	0.79	0.73	0.77
(b) Weight Imprinting	128×128	**0.90**	**0.79**	**0.84**

^*a*^ Pseudo-labeled audio segments, as explained in Section 2.3.

### 3.2. Performance on focal-recording files

We evaluated AVISNet models focal recordings corresponding to the 20% testing dataset of XC and iNat (49k audio files). For this evaluation at recording level, the model processes each file by making predictions on 3-second segments. As focal recordings ideally contain only one species, a unique final prediction is given per file, determined by summing the confidence scores across all segments and selecting the class with the highest aggregate confidence. To reduce computation – and because it has minimal impact on results – we limited inference to the first 60 seconds of each audio file. The results obtained with our models are reported in Table 2. We applied a confidence threshold of 0.5 to filter out noise segments and low confidence predictions within the file. This explains the ‘# recordings’ column in the table, which reflects the number of files with minimum one valid detection. For comparison, we include the performance of BirdNET under the same threshold. In these controlled, single-species focal recordings, AVISNet produces stable and consistent predictions, achieving performance comparable to BirdNET. However, such focal recordings do not represent realistic conditions found in the field; therefore, this evaluation mainly illustrates performance on isolated, clean vocalizations.

**Table 2:** Performance of models on weakly-labeled focal-recording files from Xeno-Canto and iNaturalist Sounds.

Model	Top-1 acc.	F1-score	# recordings
AVISNet-224	0.80	0.70	45,339
AVISNet-128	0.77	0.66	45,023
BirdNET	0.86	0.79	45,970

### 3.3. Performance on soundscape datasets

The most relevant and realistic evaluation involves real-world soundscape datasets, which capture complex acoustic environments with overlapping vocalizations from multiple species. The three soundscape datasets previously presented were employed for evaluation. Both AVISNet and BirdNET were run with a 2-second overlap to improve detection performance [27,29,32], yielding 1-second evaluation windows. For each 1-second interval, a window was considered as a positive when the target species was present within the corresponding evaluation segment (regardless of whether the vocalization occupied the entire frame or only a minor fraction of it, to prevent evaluation bias against species characterized by very short vocalizations). We considered only the top-1 model prediction (as AVISNet is a single-label multi-class classifier with softmax output), and then computed vocalization-level TP, FP and FN. Performance is summarized with macro-averaged precision (P), recall (R), and F1-score. At the dataset level, predictions were aggregated across the entire dataset to compute ecologically meaningful indicators.

Fig 5 illustrates model performance metrics as a function of the confidence threshold, evaluated from 0 to 0.95 in increments of 0.05, across the three soundscape datasets. Dataset-level *species richness* (the number of distinct species detected at least once in the dataset) and its dependence on the threshold are also illustrated. The figure shows that AVISNet requires high-confidence thresholds to avoid spurious predictions. The reason is that our models are single-label softmax classifiers, trained for yielding one prediction per input. Table 3 summarizes the best-performing results. For comparison, BirdNET results are also reported, using the recommended configuration described in Section 2.5.

**Table 3:** Evaluation metrics for three different human-annotated soundscape datasets (Th: threshold, P: precision, R: recall, F1: F1-score, TP: true positives, mAP: mean Average Precision). Best values are highlighted. Threshold selection is depicted in Fig 5.

Dataset	Model	Dataset-level					Vocalization-level				
		Th	P	R	F1	TP	Th	P	R	F1	mAP
Doñana2425^*^	AVISNet-224	*0.95*	0.43	0.80	0.56	**65**	*0.95*	0.32	0.35	0.33	0.46
	AVISNet-128	*0.95*	0.43	0.79	0.56	64	*0.95*	0.29	0.29	0.29	0.41
	BirdNET	*0.50*	0.65	0.68	**0.66**	55	*0.30*	0.46	0.58	**0.51**	**0.54**
WABAD	AVISNet-224	*0.95*	0.56	0.98	0.71	**157**	*0.60*	0.43	0.34	0.38	0.48
	AVISNet-128	*0.95*	0.58	0.97	0.72	156	*0.55*	0.39	0.32	0.35	0.47
	BirdNET	*0.50*	0.74	0.88	**0.80**	141	*0.30*	0.65	0.41	**0.51**	**0.55**
NIPS4BPlus	AVISNet-224	*0.95*	0.49	0.80	0.61	**36**	*0.90*	0.61	0.29	0.39	**0.69**
	AVISNet-128	*0.95*	0.53	0.78	0.63	35	*0.35*	0.41	0.34	0.38	0.69
	BirdNET	*0.50*	0.77	0.80	**0.78**	36	*0.30*	0.61	0.41	**0.49**	0.63

* For Doñana2425, 5-min census level instead of vocalization level was calculated.

**Fig 5:**
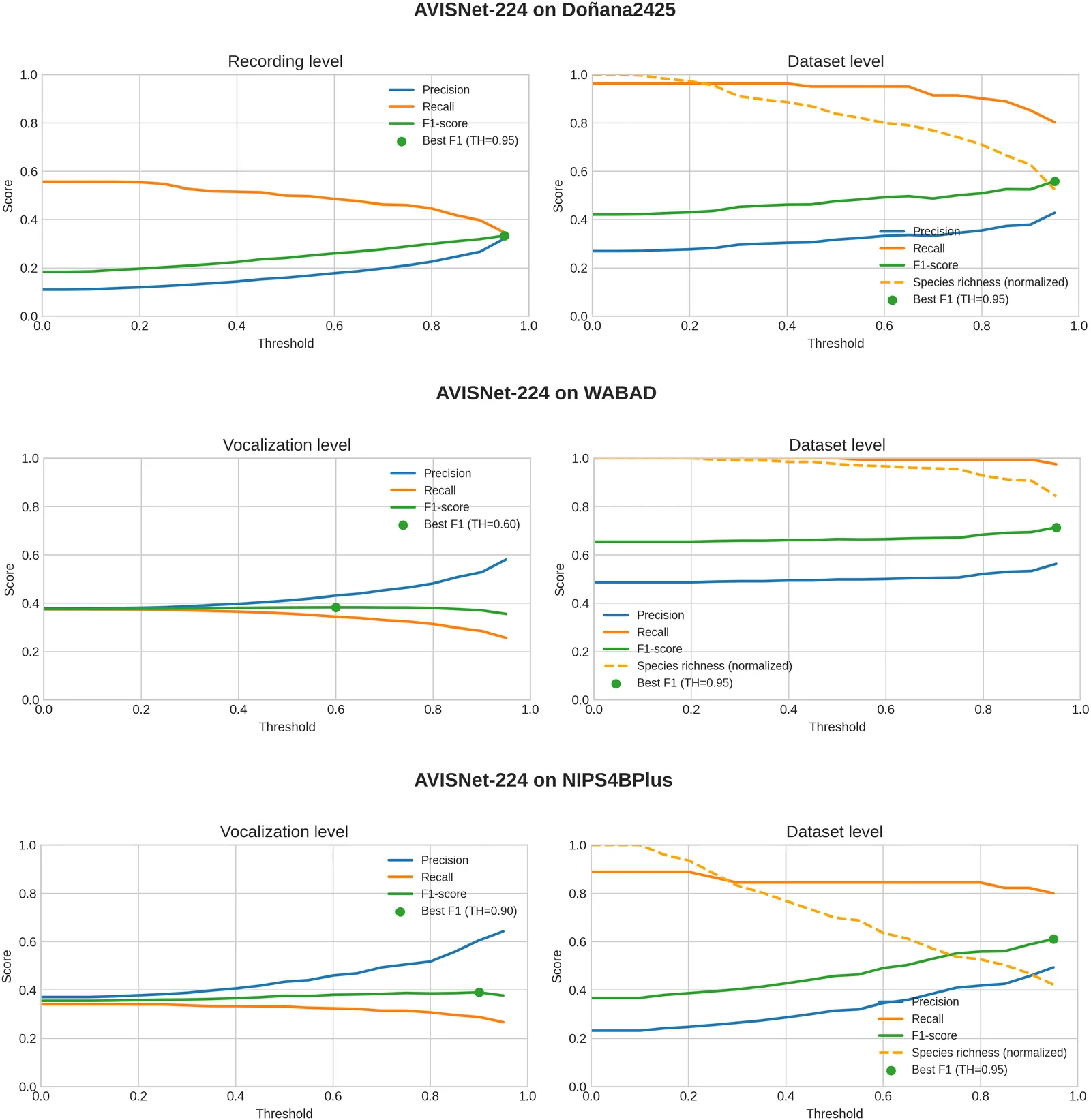
Performance metrics as function of confidence threshold for soundscape datasets. *Top*: Doñana2425; *center*: WABAD; *bottom*: NIPS4BPlus.

According to Table 3, on the Doñana2425 dataset, AVISNet achieves higher recall, detecting more correct species across datasets (dataset-level TP); while BirdNET achieves higher precision and F1-score, reflecting its larger architecture and extensive pretraining on large-scale datasets. This tendency is also observed on WABAD and NIPS4BPlus, confirming its capacity to generalize across geographically diverse habitats, and temporal and spatial scales [29]. These results highlight a complementary picture: AVISNet achieves higher recall at the dataset level, resulting in a larger correctly identified species list; while BirdNET maximizes F1-scores. Despite being much lighter than BirdNET, our models achieve dataset-level F1-scores between 0.56 and 0.72, and vocalization-level F1-scores of 0.29–0.39 with mAP values up to 0.69. AVISNet is more confident, reporting only high-confidence predictions but potentially missing lower-confidence vocalizations and thus reducing vocalization recall – rarely explores intermediate confidence values due to its single-label nature and smaller label set. BirdNET is more conservative, favoring precision and F1-score. In an edge computing paradigm operating continuously, the practical objective may shift from detecting every individual vocalization to ensuring reliable detection of species presence over longer observation periods. Because the device processes audio continuously, a species singing during a dawn chorus provides dozens of vocalization events. The probability of detecting at least one high-SNR vocalization over an extended period remains statistically high. This ‘multi-opportunity detection’ capability can partially compensate for occasional missed segments and supports the deployment of a lighter, computationally faster network that prioritize efficient long-term deployment while maintaining useful occupancy- or dataset-level performance.

Note that the choice of confidence threshold should ultimately depend on the specific ecological objective – whether maximizing species detectability (recall) or ensuring reliable identification (precision) [27]. Based on our results, we recommend using a confidence threshold of approximately 0.9 for vocalization-level analyses and a more conservative 0.95 for dataset-level evaluations using AVISNet. However, these values should be regarded as application-dependent, and further validation across more datasets will allow us to establish more reliable recommendations.

Finally, the smaller-resolution variant (AVISNet-128) maintained comparable performance to the 224 × 224 model, suggesting that resolution can be reduced to meet strict hardware constraints with reduced impact on accuracy. Model variants are publicly released for both input resolutions, trained either on the 305 well-represented classes or on the full taxonomy including under-represented species [65].

Taken together, these results indicate that AVISNet provides a practical balance between classification performance and computational efficiency for passive acoustic monitoring under real-world field conditions.

### 3.4. Hardware inference performance

AVISNet models were specifically designed with deployment efficiency in mind, targeting integration into computationally constrained audio-logging devices. Therefore, to complement accuracy-based evaluation, we benchmarked the inference efficiency.

#### 3.4.1. Embedded deployment.

To assess embedded inference performance, we measured execution times on two low-power platforms: RPi 4B and RPi Zero 2W [72,73]. RPi 4B features a quad-core 64-bit ARM Cortex-A72 CPU clocked at 1.8 GHz and 4 GB of SDRAM, while RPi Zero 2W integrates a quad-core 64-bit ARM Cortex-A53 processor running at 1 GHz with 512 MB of SDRAM and using 1 GB of swap memory. Both devices ran TensorFlow Lite (TFLite) optimized models. The evaluation was performed over 100 1-minute audio files recorded on RPi using a USB microphone. Table 4 reports average inference times and energy consumption per 1-minute recording together with relative speedups and energy reductions. It is worth mentioning that the average power consumption in idle state is 1.65 W for the RPi 4B and 0.65 W for the RPi Zero 2W.

**Table 4:** Average inference time per 1-minute audio file along with average power and energy consumption on embedded devices using TFLite. Speedups and energy reductions are relative to BirdNET.

Edge device	Model	Proc. time/file (s)	Speedup	Power (W)	Energy (J)	E. saving (%)
RPi 4B	BirdNET	6.7		3.6	24.1	
	AVISNet-224	3.0	2.2×	4.7	14.1	41.5%
	AVISNet-128	1.7	3.9×	4.0	6.8	71.8%
RPi Zero 2W	BirdNET	21.8		2.0	43.6	
	AVISNet-224	7.0	3.1×	2.8	19.6	55.0%
	AVISNet-128	4.6	4.7×	2.8	12.9	70.4%

AVISNet achieves substantial speedups – up to 4.7× faster than BirdNet – which effectively translates to operational energy savings of up to 71.8% on embedded devices, despite a small reduction in classification performance metrics. This capability of real-time species detection in the field is crucial for enabling autonomous deployments, as in edge computing, processing speedup is the direct driver of battery savings. Since total energy consumption is the integral of power over time ($E_{avg}=P_{avg}\times t_{avg}$), drastically shortening the active execution window ($t_{avg}$) – up to 4.7× faster – directly lowers the total energy consumption while also allowing the device to return to its idle state much more quickly. For instance, running AVISNet-224 on RPi Zero for analyzing one 1-minute recording demands 19.6 J (1.1 mAh), whereas BirdNET would require 43.6 J (2.4 mAh). Conversely, computationally intensive architectures lock the hardware into prolonged active states, drastically shortening battery lifespans and increasing working frequencies, which also presents second-order detrimental thermal effects [74]. Furthermore, by performing real-time classification within RAM buffers and adopting an event-driven storage scheme, the device can continuously discard audio segments in situ (e.g., those without bird activity) and only triggers physical write commands to the onboard SD card when a target biological detection occurs, thereby extending the operational lifespan of SD cards and reducing the need for manual data management and field visits [75].

Additional profiling experiments were conducted to separately measure Mel-spectrogram generation and CNN inference times per 3-second window on the target edge devices. As shown in Table 5, feature extraction accounted for approximately 23–31% of the processing time for AVISNet-224 and 40–50% for AVISNet-128, depending on the hardware platform. The remaining time corresponded to the CNN forward pass. While feature extraction represents a non-negligible fraction of the computational cost, reducing the input resolution primarily affects the CNN inference stage, decreasing its execution time from 113 ms to 50 ms on RPi 4B and from 222 ms to 102 ms on RPi Zero 2W. These results indicate that both stages contribute to the overall processing cost, although network complexity remains a major factor determining edge-device performance.

**Table 5:** Breakdown of execution time between Mel-spectrogram generation and CNN inference for AVISNet-224 and AVISNet-128 on embedded platforms. Times are reported per 3-second analysis segment.

Edge device	Model	Spectrogram (ms)	CNN (ms)
RPi 4B	AVISNet-224	34	113
	AVISNet-128	34	50
RPi Zero 2W	AVISNet-224	102	222
	AVISNet-128	101	102

**Figures of Merit** To jointly assess ecological accuracy and computational efficiency, we define the following Figures of Merit (FoM):

• The precision rate, which measures the rate of ‘reliable detections per second’:


$$\begin{array}{c} \text{Precision rate}=\frac{\text{Precision}}{t_{\text{avg}}} \end{array}$$ (1)

where *t*_avg_ represents the average inference time per second of audio in the recording.

• The recall rate, which represents the number of ‘correctly detected species per second’:


$$\begin{array}{c} \text{Recall rate}=\frac{\text{Recall}}{t_{\text{avg}}} \end{array}$$ (2)

• The efficiency score provides global measure of the trade-off between accuracy and computational cost on autonomous embedded devices as ‘model effectiveness per joule’:


$$\begin{array}{c} \text{Efficiency score}=\frac{F1\text{-score}}{t_{\text{avg}}\times P_{\text{avg}}} \end{array}$$ (3)

where *P*_avg_ stands for the average power.

Corresponding results are depicted in Fig 6, showing that AVISNet-224 achieves up to 2.4× higher precision rate than BirdNET, 3.7× higher recall rate and 2.0× efficiency score, whereas AVISNet-128 reaches 3.7×, 5.7×, and 3.1× improvements in these FoMs. These results confirm their superior trade-off between model accuracy and resource efficiency, which is crucial for autonomous embedded deployments.

**Fig 6:**
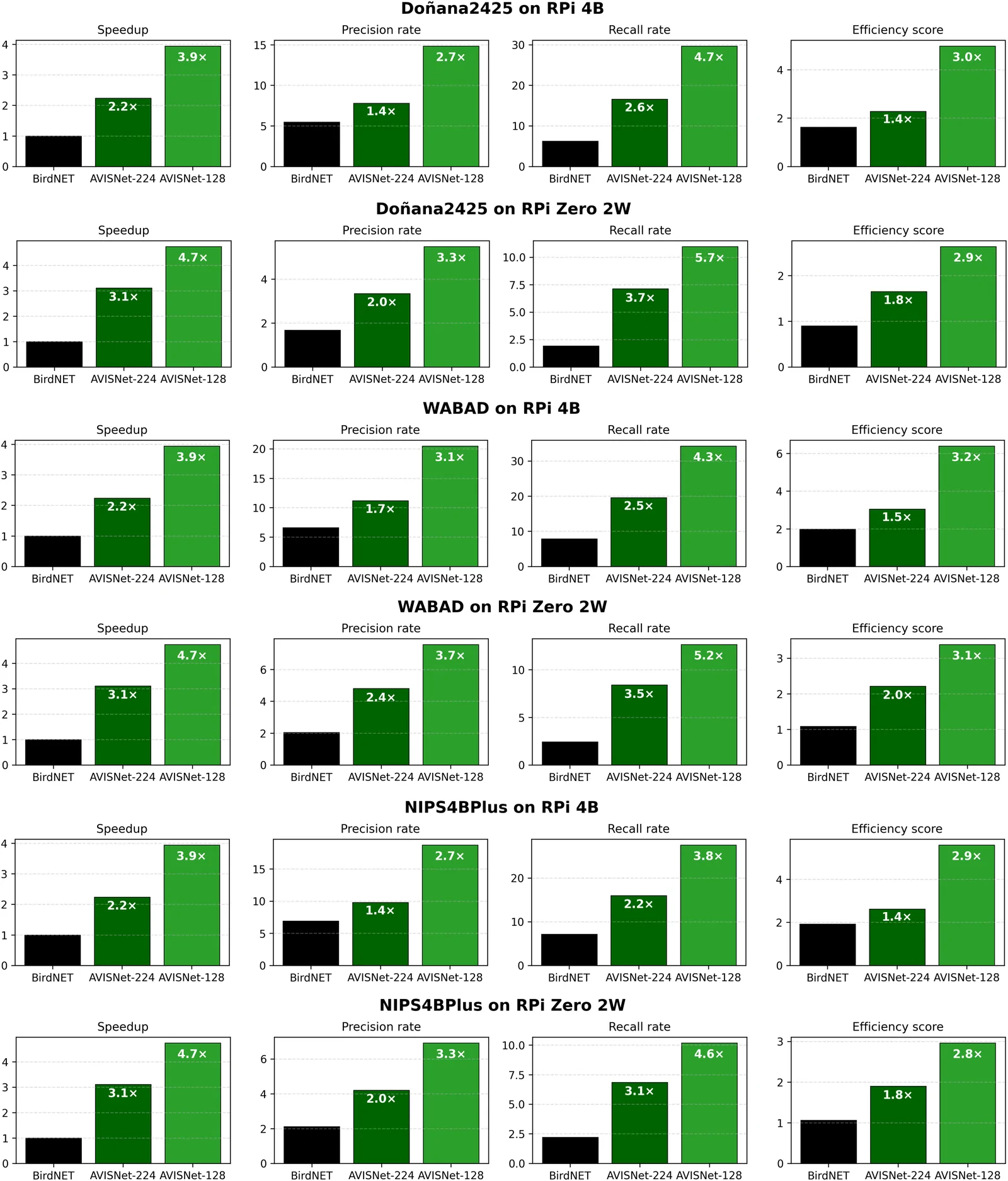
Figures of merit jointly quantifying accuracy and hardware efficiency on edge devices.

#### 3.4.2. High-end inference.

For the sake of further comparison, we also measured inference times for large-scale inference on a high-end computer running Ubuntu 22.04.5, featuring a six-core Intel i5-9600K CPU at 3.70 GHz and 32 GB of RAM. Average processing times were measured running TFLite CPU inference with a 2-second overlap for each soundscape dataset, with results are reported in Table 6. For reference, the average recording durations are 300 s for Doñana2425, 60 s for WABAD, and 4.2 s for NIPS4BPlus. Overall, these results demonstrate that AVISNet consistently surpasses BirdNET in inference speed across datasets, highlighting its suitability for large-scale or high-throughput bioacoustic processing pipelines. These performance gains highlight an emerging paradigm in computational bioacoustics: the shift from all-inclusive global models toward specialized, regional architectures [44]. While global tools like BirdNET provide remarkable multi-species coverage, they introduce a significant computational load and increase the risk of FP from geographically unfeasible species. Smaller neural networks that are custom-tuned to a specific regional community represent a highly effective path forward, delivering massive throughput accelerations not only vital for hardware-constrained edge nodes but also profoundly valuable in laboratory server environments, as demonstrated here.

**Table 6:** Inference performance on high-end computer (Ubuntu 22.04.5, 2-s overlap) for large-scale studies.

Dataset	# files	Model	proc. time/recording (s)	Speedup
Doñana2425	72 censuses	BirdNET	24.0	
		AVISNet-224	7.6	3.2×
		AVISNet-128	5.2	4.6×
WABAD	1,853 files	BirdNET	4.6	
		AVISNet-224	1.6	2.9×
		AVISNet-128	1.3	3.5×
NIPS4BPlus	687 files	BirdNET	0.3	
		AVISNet-224	0.1	3.0×
		AVISNet-128	0.1	3.0×

#### 3.4.3. Edge computing and future horizons.

The results of this study suggest that the choice between global classifiers such as BirdNET and regional edge-oriented models such as AVISNet should not be assessed solely in terms of classification accuracy. Rather, it represents a multidimensional trade-off across ecological and computational axes (Table 7). Ecologically, AVISNet specifically addresses a critical regional gap by successfully modelling 14 local bird species that are entirely absent from BirdNET’s global taxonomy. From a monitoring perspective, FN may be particularly relevant because undetected acoustic events cannot be recovered retrospectively if only detections are stored. In this context, AVISNet achieves recall values that suggest useful sensitivity for regional monitoring applications. Conversely, a FP implies writing a noisy audio segment to storage – an inconvenience easily corrected via post-processing server routines.

**Table 7:** Operational trade-offs between global models (e.g., BirdNET) and regional edge-optimized models (e.g., AVISNet).

Dimension	Global model (BirdNET)	Regional edge model (AVISNet)	Ecological & technical implications
**Taxonomic Scope**	Worldwide (~6,000 species), missing local species.	Complete regional community.	Regional models cover local gaps and eliminate predictions on geographically impossible species.
**Classifier Focus**	Superior absolute Precision and F1-score.	Lower but operationally functional performance.	BirdNET acts as a more reliable back-in-the-lab validator. AVISNet prevents critical local data loss (FP) in the field.
**Compute Profile**	Intensive CPU bound execution. Continuous high-load state.	Ultra-fast inference. Quickly back to idle state.	Edge-optimized models avoid exponential buffer accumulation and real-time data loss under continuous 100% duty cycles.
**Energy Demand**	Modest instantaneous power draw but high prolonged energy consumption per cycle.	Higher instantaneous power but reduced total energy per cycle.	Potentially longer deployments, extending ARU battery life.
**Thermal Stress**	Sustained CPU utilization risks thermal throttling in weatherproof enclosures.	Rapid execution allows the CPU to immediately revert to low-power idle states.	Prevents hardware degradation, overheating, and performance drops in outdoor field deployments.
**Storage Paradigm**	‘Indiscriminate store-process-later’ recording (heavy SD card usage).	Potential support for ‘triggered’ recording.	Shifting to triggered logging eliminates hours of ambient noise, protecting storage capacity.

Technically, the measured computational speedup and energy reduction of the proposed model enable longer battery life in battery-powered deployments. Running intensive models on ultra-low-power devices would require prolonged CPU execution windows per minute of audio. Under a continuous 100% duty cycle streaming environment, such execution profiles may increase the risk of buffer accumulation, memory overflows, and eventual data loss. Thermally, sustaining high system loads inside small, uncooled weatherproof outdoor enclosures causes severe heat accumulation, triggering thermal throttling and hardware degradation [74]. The proposed approach potentially opens a viable avenue toward event-triggered recording schemes, moving away from indiscriminate raw data logging to dramatically extend the field lifespan of ARUs [75].

Although AVISNet was primarily designed for edge deployment, its reduced computational requirements may also benefit conventional laboratory workflows involving the analysis of large PAM datasets. When ecoacoustic researchers return from seasonal monitoring campaigns with massive, uncurated audio files (often spanning thousands of recording hours), processing these datasets using global, multi-million-parameter models requires extensive server time and energy.

Beyond real-time species reporting, on-device classification may enable future adaptive monitoring strategies. For example, the detection of species of particular conservation interest could dynamically modify the recording behavior of the device, increasing recording duration, sampling frequency, or storage priority during periods of biological relevance. Such adaptive acquisition schemes could improve the capture of rare or complex vocalizations while maintaining efficient use of storage and energy resources. Exploring these closed-loop monitoring approaches represents an interesting direction for future research.

Finally, transitioning this framework from the controlled experiments reported above into fully autonomous physical hardware constitutes the immediate next step of this research line. First, future hardware iterations must benchmark the precise battery life during long-term deployment and compare it with traditional ARU operation. Second, implementing intelligent duty cycling strategies represents a key milestone. Rather than running the CNN 24/7, the field device could employ a hardware-level, low-power acoustic wake-up trigger (e.g., a simple envelope detector or signal detector) to activate the intensive CNN inference pipeline only when relevant acoustic energy is detected. Exploring these physical deployment variables will provide a technical benchmark for the next generation of smart PAM hardware.

## 4. Conclusion

This study introduces AVISNet, a lightweight neural architecture specifically designed for bird species classification at the edge, addressing both ecological and technological challenges in PAM. Recent work has highlighted a growing shift toward regionally specialized bioacoustic models [43,44], emphasizing the importance of local adaptation to species assemblages, vocal repertoires, and soundscape characteristics. In parallel, there is an increasing demand for autonomous audio-logging devices capable of on-device processing, enabling long-term monitoring in remote or resource-limited environments.

Within this context, AVISNet provides a balanced and ecologically meaningful solution. The framework combines regionally focused training with efficient embedded inference, while remaining adaptable through FSL. This design allows AVISNet to effectively incorporate under-represented and rare species, offering a scalable mechanism for extending taxonomic coverage as new data become available. Importantly, this adaptability can be achieved without compromising performance on well-represented species, a key requirement for biodiversity monitoring applications.

Across multiple soundscape datasets spanning different ecological contexts, AVISNet demonstrated competitive classification performance despite its compact architecture. While larger models such as BirdNET tend to prioritize precision, AVISNet consistently achieved high recall at the dataset level, resulting in more complete species inventories – an attribute of particular relevance for ecological surveys, community-level analyses, and conservation-oriented monitoring.

A central outcome of this work is the remarkable computational efficiency of AVISNet on embedded hardware. When deployed on low-power platforms, AVISNet achieved inference speeds up to 4.7× faster than BirdNET and energy savings up to 71%. Although this specialized model incurs a drop in absolute vocalization-level precision compared to global models, this favorable trade-off between performance and efficiency enables near real-time, on-device species detection, facilitating the selective storage or transmission of biologically relevant audio and substantially reducing data volume, power consumption, and maintenance effort. Such capabilities represent a critical step toward scalable, long-term, autonomous biodiversity monitoring in the field.

Future research on model enhancement will focus on expanding the diversity and representativeness of training data – to address our limitation of the reliance on pseudo-labeled training data, which favours relatively acoustically clean vocalizations –, leveraging additional local censuses, advanced data augmentation strategies, and synthetic audio generation approaches, including generative AI. For instance, self-supervised or large-scale audio pre-trained representations, combined with regional fine-tuning, and iterative pseudo-label refinement, could provide richer representations of low-SNR and acoustically complex vocalizations while reducing the need for exhaustive manual annotation. Second, a rigorous comparison of alternative efficient backbone architectures trained under identical datasets – including MobileNet, EfficientNet, lightweight temporal models, compact CNN-temporal hybrids and other efficient architectures – constitutes a priority for future work that may further improve feature representation while preserving low computational cost. Thirdly, conditional computation arises as a potential strategy: a very lightweight first-stage detector could identify acoustically relevant segments and activate a more complex classifier only when sufficient acoustic evidence is present. This cascaded strategy could reduce average computational and energy requirements while retaining greater capacity for challenging events. Finally, in the longer term, integrating on-device continual learning via FSL could allow deployed recorders to progressively adapt to local acoustic environments and ecological change. This perspective opens the door to intelligent, self-adapting bioacoustic monitoring systems.

## References

[1] Ceballos G, Ehrlich PR. Mutilation of the tree of life via mass extinction of animal genera. Proc Natl Acad Sci U S A. 2023;120(39):e2306987120. doi: 10.1073/pnas.2306987120 37722053 PMC10523489

[2] Underwood N, Hambäck P, Inouye BD. Large-scale questions and small-scale data: Empirical and theoretical methods for scaling up in ecology. Oecologia. 2005;145(2):177–8. doi: 10.1007/s00442-005-0057-9 15891848

[3] Sugai LSM, Silva TSF, Ribeiro JW Jr, Llusia D. Terrestrial passive acoustic monitoring: Review and perspectives. BioScience. 2018;69(1):15–25. doi: 10.1093/biosci/biy147

[4] Berger‐Tal O, Lahoz‐Monfort JJ. Conservation technology: The next generation. Conserv Lett. 2018;11(6). doi: 10.1111/conl.12458

[5] Wich SA, Piel AK. Conservation Technology. Oxford University Press; 2021.

[6] Lahoz-Monfort JJ, Magrath MJL. A comprehensive overview of technologies for species and habitat monitoring and conservation. Bioscience. 2021;71(10):1038–62. doi: 10.1093/biosci/biab073 34616236 PMC8490933

[7] Hahn NR, Bombaci SP, Wittemyer G. Identifying conservation technology needs, barriers, and opportunities. Sci Rep. 2022;12(4802). doi: 10.1038/s41598-022-08330-w PMC8938523 35314713

[8] Tuia D, Kellenberger B, Beery S, Costelloe BR, Zuffi S, Risse B, et al. Perspectives in machine learning for wildlife conservation. Nat Commun. 2022;13(1):792. doi: 10.1038/s41467-022-27980-y 35140206 PMC8828720

[9] Miao Z, Zhang Y, Fabian Z, Hernandez Celis A, Beery S, Li C, et al. New Frontiers in AI for Biodiversity Research and Conservation with Multimodal Language Models. 2024. Available from: https://ecoevorxiv.org/repository/view/7477/

[10] Speaker T, O’Donnell S, Wittemyer G, Bruyere B, Loucks C, Dancer A, et al. A global community-sourced assessment of the state of conservation technology. Conserv Biol. 2022;36(3):e13871. doi: 10.1111/cobi.13871 34904294 PMC9303432

[11] Sethi SS, Ewers RM, Jones NS, Orme CDL, Picinali L. Robust, real‐time and autonomous monitoring of ecosystems with an open, low‐cost, networked device. Methods Ecol Evol. 2018;9(12):2383–7. doi: 10.1111/2041-210x.13089

[12] Zeuss D, Bald L, Gottwald J, Becker M, Bellafkir H, Bendix J. Nature 4.0: A networked sensor system for integrated biodiversity monitoring. Conserv Biol. 2024;30(1):e17056. doi: 10.1111/gcb.17056 38273542

[13] Xie J, Zhong Y, Zhang J, Liu S, Ding C, Triantafyllopoulos A. A review of automatic recognition technology for bird vocalizations in the deep learning era. Ecol Inform. 2023;73:101927. doi: 10.1016/j.ecoinf.2022.101927

[14] Stowell D. Computational bioacoustics with deep learning: A review and roadmap. PeerJ. 2022;10:e13152. doi: 10.7717/peerj.13152 35341043 PMC8944344

[15] Napier T, Ahn E, Allen-Ankins S, Schwarzkopf L, Lee I. Advancements in preprocessing, detection and classification techniques for ecoacoustic data: A comprehensive review for large-scale Passive Acoustic Monitoring. Exp Syst Appl. 2024;252:124220. doi: 10.1016/j.eswa.2024.124220

[16] Amsalem A, Shallom ID. Time Frequency Representation for Speech Recognition. In: 2006 International Conference on Information Technology: Research and Education; 2006. pp. 99–103. doi: 10.1109/ITRE.2006.381542

[17] Cakir E, Adavanne S, Parascandolo G, Drossos K, Virtanen T. Convolutional recurrent neural networks for bird audio detection. All Open Access, Green Open Access. 2017. pp. 1744–8. doi: 10.23919/EUSIPCO.2017.8081508

[18] Xie J, Hu K, Guo Y, Zhu Q, Yu J. On loss functions and CNNs for improved bioacoustic signal classification. Ecol Inform. 2021;64:101331. doi: 10.1016/j.ecoinf.2021.101331

[19] Morgan MM, Braasch J. Open set classification strategies for long-term environmental field recordings for bird species recognition. J Acoust Soc Am. 2022;151(6):4028. doi: 10.1121/10.0011466 35778198

[20] Lasseck M. Audio-based bird species identification with deep convolutional neural networks. 2018.

[21] Research G. AudioSet. https://research.google.com/audioset/

[22] Department of Engineering Science UoO. VGG-sound. https://www.robots.ox.ac.uk/vgg/data/vggsound/

[23] Kahl S, Wood CM, Eibl M, Klinck H. BirdNET: A deep learning solution for avian diversity monitoring. Ecol Inform. 2021;61:101236. doi: 10.1016/j.ecoinf.2021.101236

[24] He K, Zhang X, Ren S, Sun J. Deep Residual Learning for Image Recognition. arXiv. 2015.

[25] Thompson MC, Ducey MJ, Gunn JS, Rowe RJ. A post‐processing framework for assessing BirdNET identification accuracy and community composition. Ibis. 2024;167(2):530–42. doi: 10.1111/ibi.13357

[26] Ware L, Mahon CL, McLeod L, Jetté J-F. Artificial intelligence (BirdNET) supplements manual methods to maximize bird species richness from acoustic data sets generated from regional monitoring. Can J Zool. 2023;101(12):1031–51. doi: 10.1139/cjz-2023-0044

[27] Funosas D, Barbaro L, Schillé L, Elger A, Castagneyrol B, Cauchoix M. Assessing the potential of BirdNET to infer European bird communities from large-scale ecoacoustic data. Ecol Indic. 2024;164:112146. doi: 10.1016/j.ecolind.2024.112146

[28] Scanferla J, Brambilla M, Brambilla G, Hilpold A, Marchetti AE, Puff F, et al. Determining species-specific thresholds to improve precision in passive acoustic monitoring. Ecol Inform. 2025;91:103423. doi: 10.1016/j.ecoinf.2025.103423

[29] Fairbairn AJ, Burmeister J-S, Weisser WW, Meyer ST. BirdNET can be as good as experts for acoustic bird monitoring in a European city. PLoS One. 2025;20(9):e0330836. doi: 10.1371/journal.pone.0330836 40934194 PMC12425287

[30] Manzano-Rubio R, Bota G, Brotons L, Soto-Largo E, Pérez-Granados C. Low-cost open-source recorders and ready-to-use machine learning approaches provide effective monitoring of threatened species. Ecol Inform. 2022;72:101910. doi: 10.1016/j.ecoinf.2022.101910

[31] Pérez‐Granados C. BirdNET: applications, performance, pitfalls and future opportunities. Ibis. 2023;165(3):1068–75. doi: 10.1111/ibi.13193

[32] Pérez-Granados C, Funosas D, Morant J, Marín Gómez OH, Mendoza I, Mohedano-Munoz MA. Optimization of passive acoustic bird surveys: A global assessment of BirdNET settings. Ibis. 2023. doi: 10.1111/ibi.70013

[33] Wood CM, Kahl S. Guidelines for appropriate use of BirdNET scores and other detector outputs. J Ornithol. 2024;165(3):777–82. doi: 10.1007/s10336-024-02144-5

[34] Williams B, van Merrienboer B, Dumoulin V, Hamer J, Triantafillou E, Fleishman AB, et al. Leveraging tropical reef, bird and unrelated sounds for superior transfer learning in marine bioacoustics. ArXiv. 2024. Available from: https://api.semanticscholar.org/CorpusID:269362674 10.1098/rstb.2024.0280 PMC12159673 40501129

[35] McGinn K, Kahl S, Peery MZ, Klinck H, Wood CM. Feature embeddings from the BirdNET algorithm provide insights into avian ecology. Ecol Inform. 2023;74:101995. doi: 10.1016/j.ecoinf.2023.101995

[36] Ghani B, Denton T, Kahl S, Klinck H. Global birdsong embeddings enable superior transfer learning for bioacoustic classification. Sci Rep. 2023;13(1):22876. doi: 10.1038/s41598-023-49989-z 38129622 PMC10739890

[37] Vandendriessche J, Wouters N, da Silva B, Lamrini M, Chkouri MY, Touhafi A. Environmental sound recognition on embedded systems: From FPGAs to TPUs. Electronics. 2021;10(21):2622. doi: 10.3390/electronics10212622

[38] Höchst J, Bellafkir H, Lampe P, Vogelbacher M, Mühling M, Schneider D, et al. Bird@Edge: Bird Species Recognition at the Edge. In: Proceedings of 10th International Conference on Networked Systems (NETYS). Berlin, Heidelberg: Springer; 2022. pp. 69–86. doi: 10.1007/978-3-031-17436-0_6

[39] Vidaña-Vila E, Navarro J, Borda-Fortuny C, Stowell D, Alsina-Pagès RM. Low-cost distributed acoustic sensor network for real-time urban sound monitoring. Electronics. 2020;9(12):2119. doi: 10.3390/electronics9122119

[40] Shah SK, Tariq Z, Lee Y. IoT based Urban Noise Monitoring in Deep Learning using Historical Reports. In: Proceedings of IEEE International Conference on Big Data (Big Data). 2019. pp. 4179–84. doi: 10.1109/BigData47090.2019.9006176

[41] Delgado-Rajó FA, Travieso-Gonzalez CM. Flexible hybrid edge computing IoT architecture for low-cost bird songs detection system. Ecol Inform. 2025;90:103231. doi: 10.1016/j.ecoinf.2025.103231

[42] da Silva B, Happi W, Braeken A, Touhafi A. Evaluation of classical machine learning techniques towards urban sound recognition on embedded systems. Appl Sci. 2019;9(18):3885. doi: 10.3390/app9183885

[43] Lauha P, Somervuo P, Lehikoinen P, Geres L, Richter T, Seibold S, et al. Domain‐specific neural networks improve automated bird sound recognition already with small amount of local data. Methods Ecol Evol. 2022;13(12):2799–810. doi: 10.1111/2041-210x.14003

[44] Huus J, Kelly KG, Bayne EM, Knight EC. HawkEars: A regional, high-performance avian acoustic classifier. Ecol Inform. 2025;87:103122. doi: 10.1016/j.ecoinf.2025.103122

[45] Márquez-Rodríguez A, Mohedano-Munoz MÁ, Marín-Jiménez MJ, Santamaría-García E, Bastianelli G, Jordano P, et al. A bird song detector for improving bird identification through deep learning: A case study from Doñana. Ecol Inform. 2025;90:103254. doi: 10.1016/j.ecoinf.2025.103254

[46] para la Transición Ecológica y el Reto Demográfico M. Parque Nacional de Doñana. 2024. https://www.miteco.gob.es/es/parques-nacionales-oapn/red-parques-nacionales/parques-nacionales/donana.html

[47] Foundation Center NB. xeno-canto. Sharing wildlife sounds from around the world. https://xeno-canto.org/

[48] Tivirikin N. xeno-canto API Wrapper. https://github.com/ntivirikin/xeno-canto-py

[49] Chasmai M, Shepard A, Maji S, Van Horn G. The iNaturalist Sounds Dataset. Advances in Neural Information Processing Systems. 2024.

[50] Piczak KJ. ESC: Dataset for Environmental Sound Classification. In: Proceedings of the 23rd Annual ACM Conference on Multimedia. ACM Press;. pp. 1015–8. Available from: http://dl.acm.org/citation.cfm?doid=2733373.2806390

[51] Turpault N, Serizel R, Vincent E. Analysis of weak labels for sound event tagging. hal-03203692. 2021.

[52] Sprengel E, Jaggi M, Kilcher Y, Hofmann T. Audio Based Bird Species Identification using Deep Learning Techniques. In: Conference and Labs of the Evaluation Forum (CLEF). 2016. pp. 547–59. Available from: http://ceur-ws.org/Vol-1609/16090547.pdf

[53] Lasseck M. Improving Bird Recognition using Pseudo-Labeled Recordings from the Target Location. In: Conference and Labs of the Evaluation Forum; 2024. Available from: https://api.semanticscholar.org/CorpusID:271866079

[54] Miyaguchi A, Cheung A, Gustineli M, Kim A. Transfer Learning with Pseudo Multi-Label Birdcall Classification for DS@GT BirdCLEF 2024. arXiv. 2024. Available from: https://arxiv.org/abs/2407.06291

[55] Lee DH. Pseudo-label: The simple and efficient semi-supervised learning method for deep neural networks. Workshop on Challenges in Representation Learning. ICML; 2013.

[56] scikit-maad: Soundscape analysis in Python. https://scikit-maad.github.io/

[57] Abadi M, et al. TensorFlow: A system for large-scale machine learning. In: 12th USENIX Symposium on Operating Systems Design and Implementation (OSDI 16). 2016. pp. 265–83.

[58] Howard A, Zhu M, Chen B, Kalenichenko D, Wang W, Weyand T, et al. MobileNets: Efficient Convolutional Neural Networks for Mobile Vision Applications. arXiv. 2017.

[59] Sandler M, Howard AG, Zhu M, Zhmoginov A, Chen L. Inverted Residuals and Linear Bottlenecks: Mobile Networks for Classification, Detection and Segmentation. arXiv. 2018.

[60] Hinton G, Vinyals O, Dean J. Distilling the Knowledge in a Neural Network. arXiv. 2015. Available from: https://arxiv.org/abs/1503.02531

[61] Parnami A, Lee M. Learning from Few Examples: A Summary of Approaches to Few-Shot Learning. arXiv. 2022. Available from: https://arxiv.org/abs/2203.04291

[62] Snell J, Swersky K, Zemel RS. Prototypical Networks for Few-shot Learning. arXiv. 2017. Available from: http://arxiv.org/abs/1703.05175

[63] Hosoda K, Nishida K, Seno S, Mashita T, Kashioka H, Ohzawa I. It’s DONE: Direct ONE-shot learning with quantile weight imprinting. arXiv. 2022. Available from: https://arxiv.org/abs/2204.13361

[64] Qi H, Brown M, Lowe DG. Low-Shot Learning with Imprinted Weights. arXiv. 2018. Available from: https://arxiv.org/abs/1712.07136

[65] Velasco-Montero D. DonanaAcousticModel. https://github.com/DVM000/DonanaAcousticModel/

[66] van Merriënboer B, Hamer J, Dumoulin V, Triantafillou E, Denton T. Birds, bats and beyond: evaluating generalization in bioacoustics models. Front Bird Sci. 2024;3. doi: 10.3389/fbirs.2024.1369756

[67] Bastianelli G. Doñana2425, 5-minute censuses dataset. Zenodo; 2025. Available from: https://zenodo.org/uploads/17732103

[68] Bibby CJ, Burgess ND, Hill DA, Mustoe SH. Bird census techniques. 2nd ed. Academic Press; 2000.

[69] Leu M, Farnsworth ML, Fleishman E, Dobkin DS, Scherer RD, Noon BR, et al. Effects of point‐count duration on estimated detection probabilities and occupancy of breeding birds. J Field Ornithol. 2017;88(1):80–93. doi: 10.1111/jofo.12183

[70] et al CPG. WABAD: A world annotated bird acoustic dataset for passive acoustic monitoring. Res Square. 2025. doi: 10.21203/rs.3.rs-5729784/v1 PMC12881925 41652929

[71] Morfi V, Bas Y, Pamuła H, Glotin H, Stowell D. NIPS4Bplus: A richly annotated birdsong audio dataset. PeerJ Comput Sci. 2019;5:e223. doi: 10.7717/peerj-cs.223 33816876 PMC7924541

[72] Raspberry Pi 4. https://www.raspberrypi.com/products/raspberry-pi-4-model-b/

[73] Raspberry Pi Zero 2W. https://www.raspberrypi.com/products/raspberry-pi-zero-2-w/

[74] Benoit-Cattin T, Velasco-Montero D, Fernández-Berni J. Impact of thermal throttling on long-term visual inference in a CPU-based edge device. Electronics. 2020;9(12):2106. doi: 10.3390/electronics9122106

[75] Benhamadi Y, Bernabeu A, Morandi I, Kwak M, Lostanlen V, et al. Modeling Battery Lifetime in TinyML-enabled Bioacoustic Surveys: A case study with the AudioMoth sensor and Yellowhammer (Emberiza citrinella). HAL; 2026. Available from: https://hal.science/hal-05618450

